# DNA-based customized functional modules for signal transformation

**DOI:** 10.3389/fchem.2023.1140022

**Published:** 2023-02-14

**Authors:** Mingzhi Zhang, Yang Sun

**Affiliations:** Institute of Molecular Medicine (IMM), Renji Hospital, State Key Laboratory of Oncogenes and Related Genes, Shanghai Jiao Tong University School of Medicine, Shanghai, China

**Keywords:** DNA functional modules, molecular sensing, DNA computing, signal transformation, DNA network

## Abstract

Information on the temporal and spatial scale of cellular molecules in biological systems is crucial for estimating life processes and may be conducive to an improved understanding of disease progression. This intracellular and extracellular information is often difficult to obtain at the same time due to the limitations of accessibility and sensing throughput. DNA is an excellent material for *in vivo* and *in vitro* applications, and can be used to build functional modules that can transform bio-information (input) into ATCG sequence information (output). Due to their small volume and highly amenable programming, DNA-based functional modules provide an opportunity to monitor a range of information, from transient molecular events to dynamic biological processes. Over the past two decades, with the advent of customized strategies, a series of functional modules based on DNA networks have been designed to gather different information about molecules, including the identity, concentration, order, duration, location, and potential interactions; the action of these modules are based on the principle of kinetics or thermodynamics. This paper summarizes the available DNA-based functional modules that can be used for biomolecular signal sensing and transformation, reviews the available designs and applications of these modules, and assesses current challenges and prospects.

## 1 Introduction

Biological life is one of the most complex and dynamic systems in nature. In order to survive and reproduce, cells must sense a wide variety of inputs, both external and internal, and must compute and actuate a number of output signals (Hartwell et al., 1999). These biotic signals, including ions, metabolites, nucleic acids, or proteins, are manipulated into cell activity and functions (Masel and Siegal, 2009; Antebi et al., 2017). Therefore, monitoring the physicochemical, structural, and spatial properties of biologically active molecules is significant for studying biological systems and processes (Purvis and Lahav, 2013).

**FIGURE 1:**
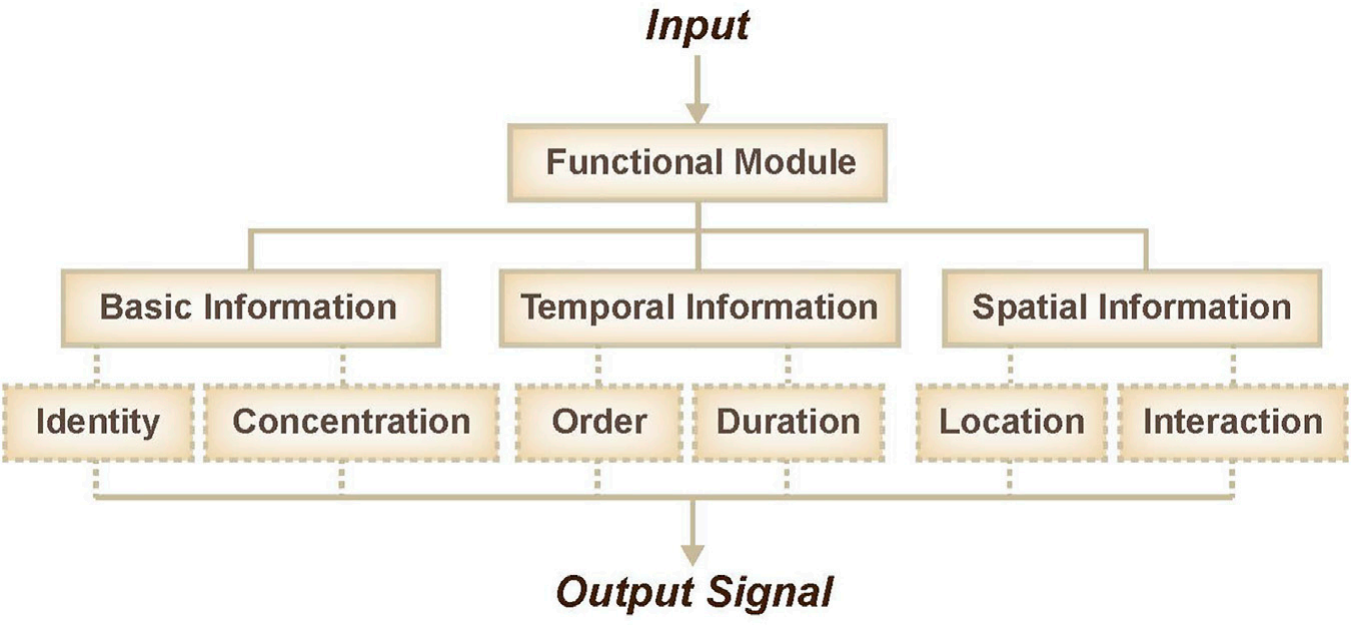
Overview of functional modules, including basic information sensing, temporal information sensing, and spatial information sensing.

DNA is an important biomacromolecule that stores and transfers genetic information in a cell; it is made up of four chemical bases: adenine (A), guanine (G), cytosine (C), and thymine (T). DNA is a well-known nanoscale engineering material due to its highly amenable programming and storage capacities. Due to the predictability of its double-helical structure and its Watson-Crick binding thermodynamics, remarkably complex static structures that self-assemble can be rationally designed from synthetic DNA oligonucleotides of defined sequences (Zhang and Winfree, 2009). Due to its good biocompatibility and small volume, DNA is an excellent candidate for *in vivo* and *in vitro* information sensing and integration. Over the past few decades, DNA networks have exhibited great performance in recognition (Zhou et al., 2020), classification (Lopez et al., 2018; Zhang et al., 2020), and information integration (Su et al., 2019; Zhao et al., 2021). With the advantages of standardization and modularization, DNA-based functional modules have inspired plausible ideas to achieve specific goals. Historically, the approach to the sensing of molecular information has been based on bespoke, unique solutions. Various strategies can be used to couple the activity of a given functional module to signals of interest. For example, sensors or switches have been used to transform molecular identity information into oligonucleotides, thresholds or selectors have been used to transform concentration information, sequencers or selectors have been used to transform order information, time-delay units (including accumulation of delay signal or consumption of timer) have been used to transform duration information, and various other techniques have been used to transform molecular information like location and potential interactions (Figure 1).

In this review, recent progress in the emerging field of DNA-based functional modules is discussed. Key elements in the strategies underlying different molecular information sensing are highlighted and several approaches based on the same principle are described. This paper ends with a description of the current technical challenges and knowledge gaps, the possible applications of these functional modules, and speculation as to the future of this exciting area of research and development.

## 2 Strategies for functional modules

A variety of signals, including small molecules, RNA, proteins, electricity, heat, and light, can be converted from DNA signals, providing an excellent interface between biological and non-biological systems (Sheth and Wang, 2018). These signals can be transformed into oligo format and used by downstream modules based on the DNA strand-displacement reaction. The DNA strand-displacement reaction is a dynamic molecular process between a single strand and a complementary double strand. In this reaction, an invaded strand serves as the input, while a double-strand complex serves as the convertor. The invaded strand will replace and release the constrained single strand *via* branch migration in the original double strand, thereby generating a more thermodynamically stable double-stranded structure; then, the released single strand can serve as the output (Zhang and Winfree, 2009).

### 2.1 Basic information sensing

The identity and concentration of molecules are basic pieces of information that are of interest in a cell. Since different molecules often serve different functions, identity is critical for analyzing biological processes. Furthermore, changes in concentration usually relate to function execution and state transition. Biologists can use biotechnologies to quantify proteins or metabolites in order to monitor changes in biological processes. Therefore, basic information sensing is crucial for evaluating life processes and performing clinical diagnostics. Several methods have been developed for basic molecular information sensing, and the strategies based on DNA functional modules are particularly significant within this field (Zhou et al., 2020).

#### 2.1.1 Molecule identification

DNA, RNA, proteins, and metabolites are key molecules in life processes. These types of molecules have different significance in a biosystem. Based on their different physicochemical properties, two types of devices have been used for identity sensing: sensors and switches. Sensors sense information with analog signals while switches transform this information into digital format. In DNA-based sensors and switch circuits, information must be transformed into a standardized format, for example, oligo format (Sheth and Wang, 2018).

**FIGURE 2:**
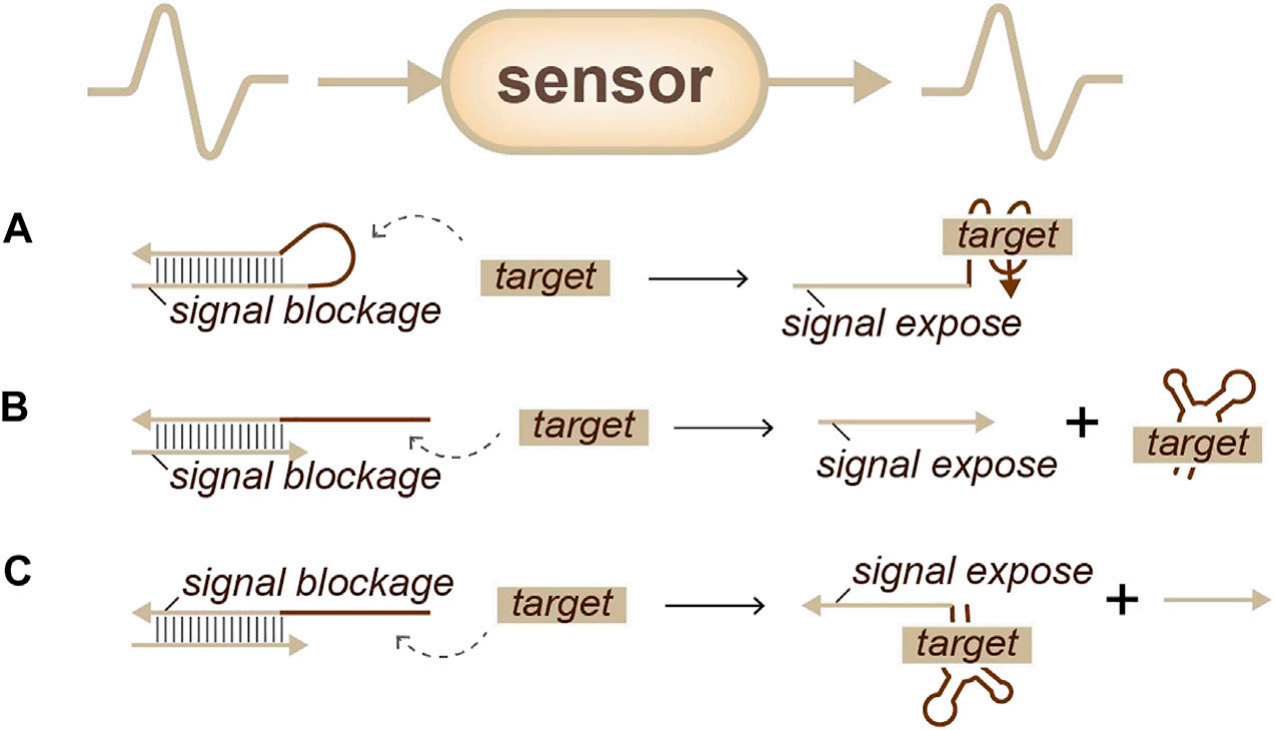
Strategies for identity sensing, sensors: analog information can be transferred into analog signals. **(A)** An aptamer-beacon (Hamaguchi et al., 2001): signals can be exposed when a target appears. **(B)** An aptamer-heterodimer (Nutiu and Li, 2003): signals can be released when a target appears. **(C)** An aptamer-heterodimer (Nutiu and Li, 2004): signals can be exposed when a target arrives.

In the early 2000s, researchers attempted to use aptamers, which are short DNA or RNAs that can specifically bind to a target such as an antibody, for this purpose. Aptamers can trap molecules and expose or release a recognizable sequence to achieve information transformation (Hamaguchi et al., 2001; Nutiu and Li, 2003; Nutiu and Li, 2004). There are two types of aptamer strategies. The first is closed-state aptamer beacons (Figure 2A): when the target appears, the beacon interacts with the target, uncoiling and forming a new structure that exposes a special domain (Hamaguchi et al., 2001). The second is heterodimers (Figures 2B, C), which contain one aptamer and the complementary auxiliary signal strand. Once the target appears, the aptamer responds and releases the signal strand (Nutiu and Li, 2003; Nutiu and Li, 2004). From these two strategies, several variants have been designed. For example, to reduce signal leakage, Wang’s group developed a molecular aptamer beacon-tuned DNA strand displacement reaction. In this work, the strand displacement mode was used to transform adenosine triphosphate (ATP) input into a DNA strand output signal for the downstream gates to process (Zhu et al., 2014). Moreover, Katz’s group proposed that enzymes can be sensed through released oligos caused by enzyme functional reactions (Mailloux et al., 2015).

**FIGURE 3:**
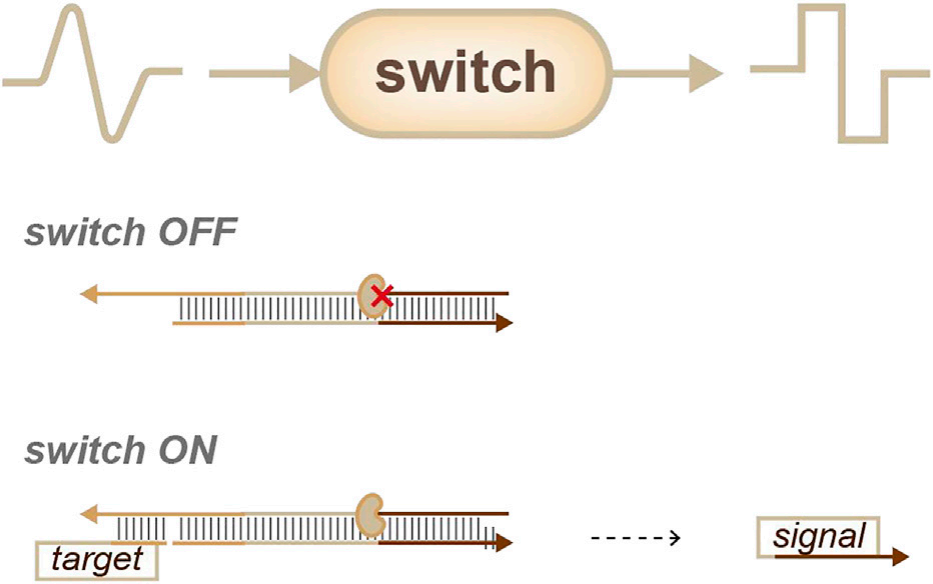
Strategies for identity sensing, switches (Kim et al., 2006): analog information can be transferred into digital signals.

Though switches (Figure 3) are not as common as sensors, they are still an important means of identity sensing. In 2006, Winfree’s group reported a strategy called “genlet”. In this strategy, a target can be linked to the enzyme or the function domain of the genlet, so that target information can be transformed into oligos through the genlet. Once the target is identified, the promoter is activated and produces a new sequence with the function of the enzyme. This serves as a standardized device to form a complex circuit (Kim et al., 2006). Likewise, Ricci’s group described a transcriptional switch module for the detection of specific target antibodies. In this work, a programmable antigen was conjugated to DNA-based conformational switches (antibody-responsive transcriptional switches). The binding of the antigen to the antibody triggered the cell-free transcription of an RNA aptamer (Patino Diaz et al., 2022). These cell-free transcriptional switches can efficiently and directly measure antibody levels in blood serum and might have good application prospects for diagnostic tests.

In short, great progress has been made in the use of DNA functional modules to sense basic molecular information. The use of these molecules for information sensing is of great significance for fundamental science and clinical transformation.

#### 2.1.2 Concentration estimation

The concentration of a molecule is important information that provides insight into biological processes. The upregulation and downregulation of an element in a pathway usually causes different phenotypes. Scientists have long used a variety of methods to obtain information about the concentration of various molecules, e.g., standard curves, ELISA, western blot, real-time PCR, and even sequencing. Moreover, various novel strategies that use DNA networks for concentration estimation have been developed.

**FIGURE 4:**
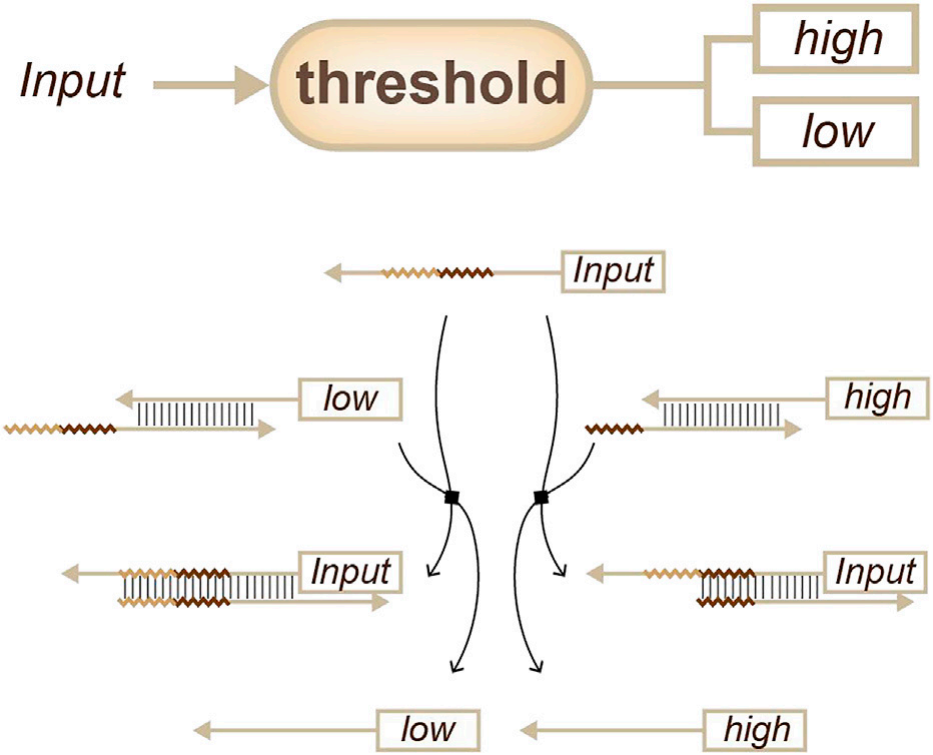
Strategies for concentration sensing, a threshold that more than one signal can be output. (Qian and Winfree, 2011).

In the late 2000s, Zhang, Y. and Winfree, E*.* proposed a simple kinetics theory for toehold-mediated strand displacement. This theory holds that kinetics can be accurately modelled and predicted from the length and sequence of the toehold domain (Zhang and Winfree, 2009). Based on this theory, a multitude of concentration-related devices were developed that could be scaled up into large networks. The most successful application was the development of thresholds (Figure 4) for different concentrations (Qian and Winfree, 2011). With such thresholds, a complex network, such as a neural computation network, can be built to achieve pattern recognition (Qian et al., 2011; Cherry and Qian, 2018).

**FIGURE 5:**
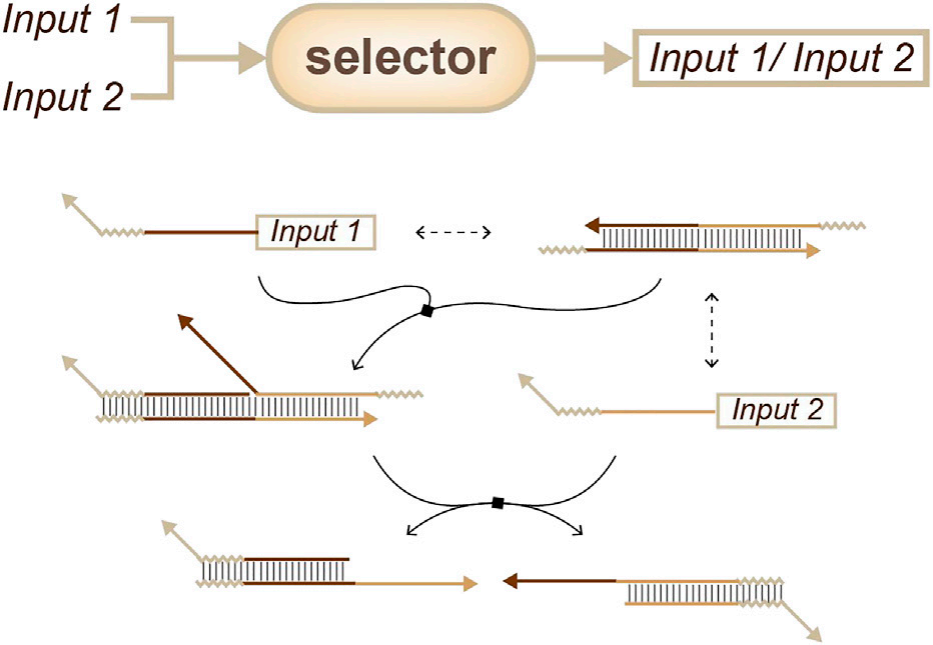
Strategies for concentration sensing, a selector that only one signal can be output. (Cherry and Qian, 2018; Rodriguez et al., 2021).

For simple concentration estimation, thresholds can be used to obtain outputs with different concentrations (Han et al., 2012). For example, the concentration of a target can be sensed and tuned. Furthermore, strategies such as “winner-take-all” or “loser-take-all” can be chosen to obtain only one output from the side of interest (Figure 5) (Cherry and Qian, 2018; Rodriguez et al., 2021).

### 2.2 Temporal information sensing

Temporal information includes the order of appearance and the duration. Temporal information is significant in embryonic development and etiological diagnoses. Neurons use the relative timing of synaptic spikes to modify learning while cells use the relative timing of transcription factors to respond to stress. The relative timing of signals is universal and temporal information processing is powerful. Several strategies have been developed for temporal information sensing, such as dynamic visualization, current monitoring, and single-cell sequencing, among others. These strategies are often used to monitor or trace time-dependent changes in a target molecule. While the available strategies are not direct or continuous sensing strategies, with the further development of theories and practices in DNA functional modules, the tracing of temporal information may become more direct and continuous.

#### 2.2.1 Order identification

**FIGURE 6:**
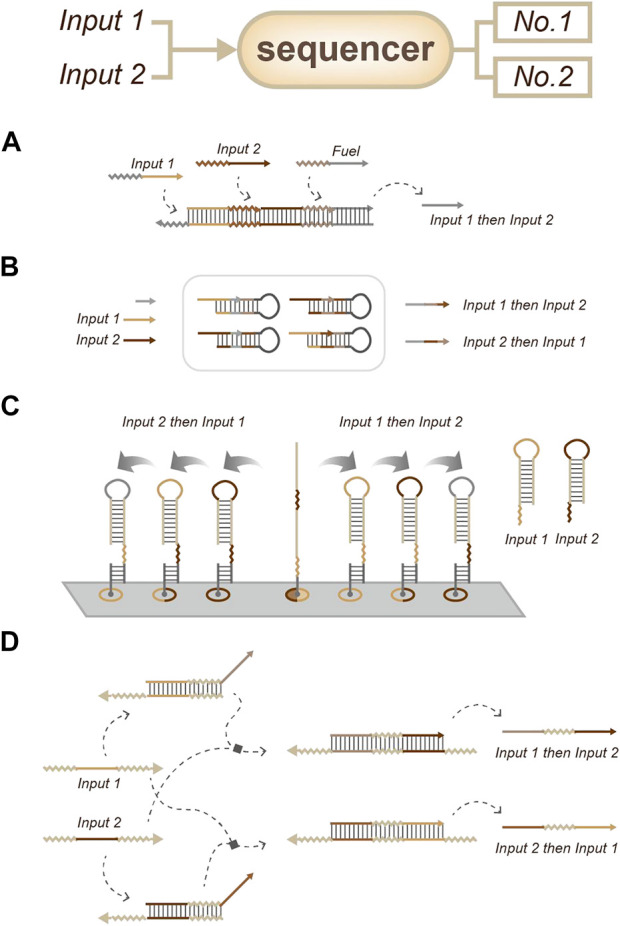
Strategies for order sensing, a sequencer that all order information can be readout from the output signals. **(A)** Temporal memory. (Lakin and Stefanovic, 2017). **(B)** Primer exchange reaction (PER) for event recording. (Kishi et al., 2018). **(C)** Approach based on the energy landscape. (Liu et al., 2022). **(D)** Approach based on strand-displacement circuits. (Lapteva et al., 2022).

In most situations, order information can be inferred by the relative time information. There are two common strategies to detect order: sequencer (sequence all the targets) and selector (only output the first target). As a sequencer-related strategy, Matthew R. Lakin and Darko Stefanovic developed a strategy called temporal memory. This strategy does not yield any circuit output until all input signals have arrived; the earlier inputs will lead to distinct circuit responses to later inputs. Therefore, this strategy is suitable for combinatorial regulation based on the sequence of a small number of inputs (Figure 6A) (Lakin and Stefanovic, 2017). In 2018, Yin’s lab used a primer exchange reaction (PER) to record sequential events (Figure 6B) (Kishi et al., 2018). In 2022, Yan’s lab developed an approach based on the facile modulation of the energy landscape (different inputs with different reaction energies lead to different reaction orders) to achieve order sensing and output (Figure 6C) (Liu et al., 2022). In the same year, Qian’s group described an order-access strategy for temporal memory in DNA-only systems (Figure 6D). In this work, they used simple, two-stranded gate motifs to sense temporal memory. This strategy is not only compatible with combinatorial regulation but also enables temporal information to be incorporated into Boolean logic computation. This strategy appears to be crucial for the scalability of DNA strand-displacement circuits due to its robustness to synthesis errors and structural malformation (Lapteva et al., 2022).

**FIGURE 7:**
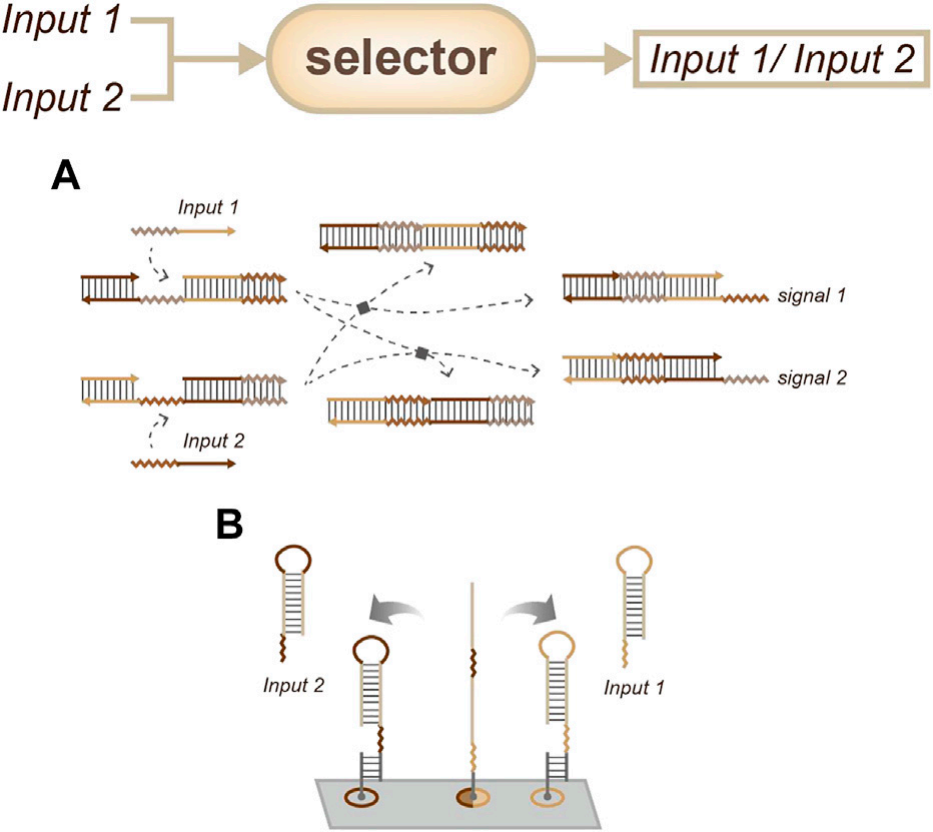
Strategies for order sensing, a selector that only outputs the former input. **(A)** Approach based on cross-inhibition. (Liu et al., 2020). **(B)** Approach based on the energy landscape. (Liu et al., 2022).

In contrast, the selector strategy chooses to only output the first input. There are two main selector strategies: the first is cross-inhibition and the second is based on the energy landscape, which is similar to the method described above. Cross inhibition produces output when the first input signal arrives, while also inhibiting the interaction between the devices and the target subsequent inputs (Figure 7A) (Liu et al., 2020). On the other hand, energy landscape strategies (Figure 7B) will not inhibit these interactions, and in some cases, these strategies may output two types of information at the same time when two targets are input in a narrow time window (Liu et al., 2022).

#### 2.2.2 Time of duration

A time-relative device is necessary for estimating duration information. A suitable duration sensing module must have a steady time interval. A delay gate is suitable for this task, as it will allow output signal release after a set time. Delay devices have been designed based on two different principles: delay signal accumulation or timer consumption.

**FIGURE 8:**
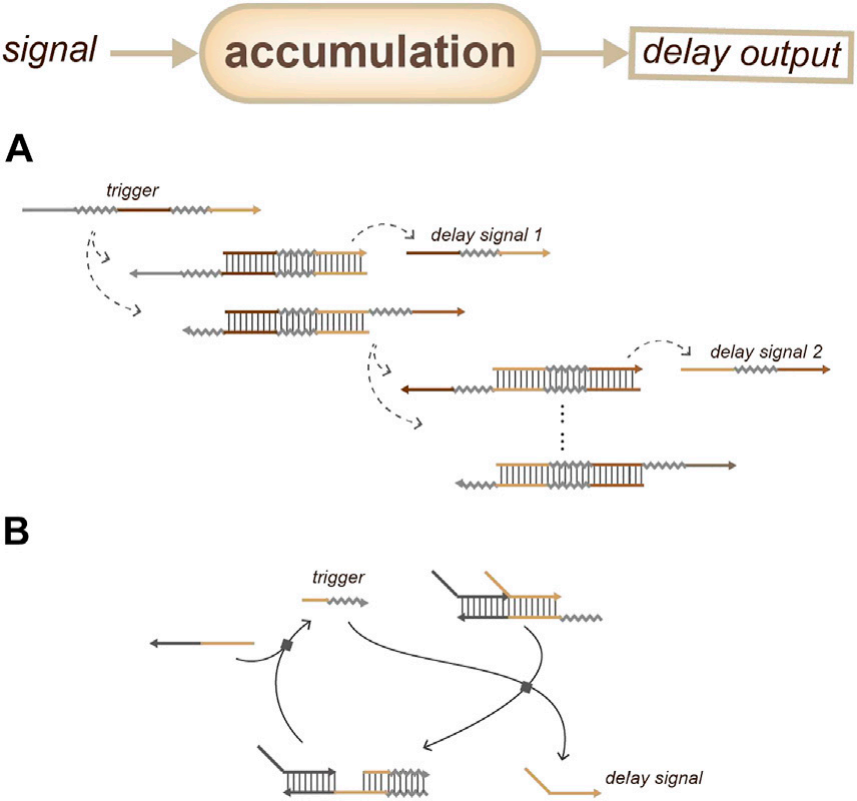
Strategies for duration sensing which based on the accumulation of delay signals. **(A)** Time delay depends on the accumulation of reaction steps. (Scalise et al., 2020). **(B)** Time delay depends on the initial concentration of the trigger. (Zhang et al., 2007).

For accumulation-based delay devices, one strategy is based on coupled biochemical reactions (Figure 8A): the delay gate can be achieved by releasing a series of different short DNA sequences one after another as the controllable delay signals; this depends on a reaction step in the output device (Scalise et al., 2020). Another strategy (Figure 8B) involves the building of a circular reaction with fuel: the delay time depends on the initial concentration of the trigger (Zhang et al., 2007).

**FIGURE 9:**
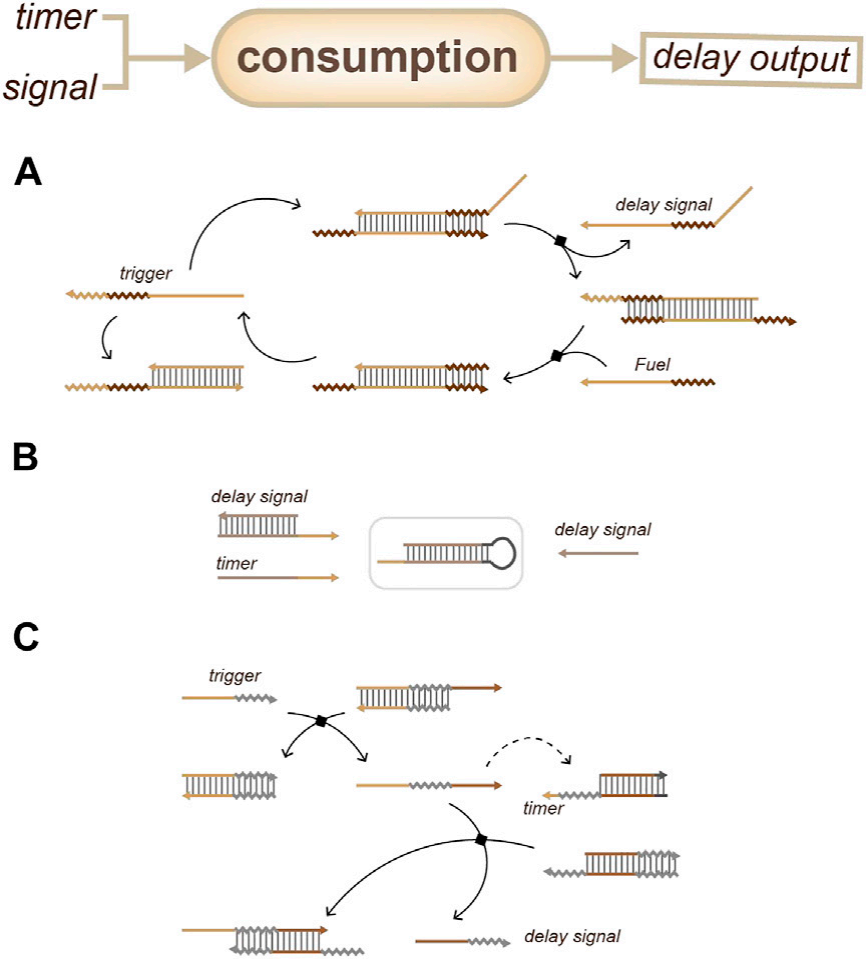
Strategies for duration sensing which based on timer consumption. **(A)** Delay signal can be output when the threshold device is exhausted. (Qian and Winfree, 2011). **(B)** Delay signal can be output when the timer is exhausted. (Aubert et al., 2014). **(C)** The trigger will release a single strand through a strand displacement reaction, which will participate in the reaction with the timer and the delay device. The delay signal will be output when the timer is exhausted. (Fern et al., 2016).

For consumption-based delay devices, one strategy (Figure 9A) is to couple a threshold and an amplifier: the threshold will postpone the signal response and the amplifier will accelerate the accumulation of the delay signal output (Qian and Winfree, 2011). Another strategy (Figure 9B, C) is to set up a defined reaction that has a rigid higher reaction priority and a fixed time. Once the reaction is over, the output can respond, like a time-lapse response (Aubert et al., 2014; Fern et al., 2016). These strategies are all based on the differences in the kinetics of relative reactions to achieve an output delay.

### 2.3 Spatial information sensing

The spatial information of molecules, i.e., the location of, for example, receptors on the cell membrane or transcription effectors in the nuclei, is important for understanding molecular functions. With decades of development, there have been great advances in the techniques for molecular spatial mapping and target discrimination. Such techniques include confocal microscopy, western blot, and spatial transcriptome. In this section, strategies for measuring the distance between two different positions and identifying potential interactions between targets will be discussed.

#### 2.3.1 Composition and spatial organization

Molecules on the cell membrane are dynamic and play an important role in the response of the cell to external stimuli. Therefore, studying the composition and spatial organization of molecules on the cell membrane is necessary for understanding biological processes. In order to obtain this information on tiny structures, DNA nanotubes have been used to connect pairs of molecular landmarks with different distances and relative orientations. The nanotubes can then join end to end to form stable connections, with unconnected nanotubes selectively melting away. These connections are separated by only 1–10 µm in more than 75% of cases (Mohammed et al., 2017).

**FIGURE 10:**
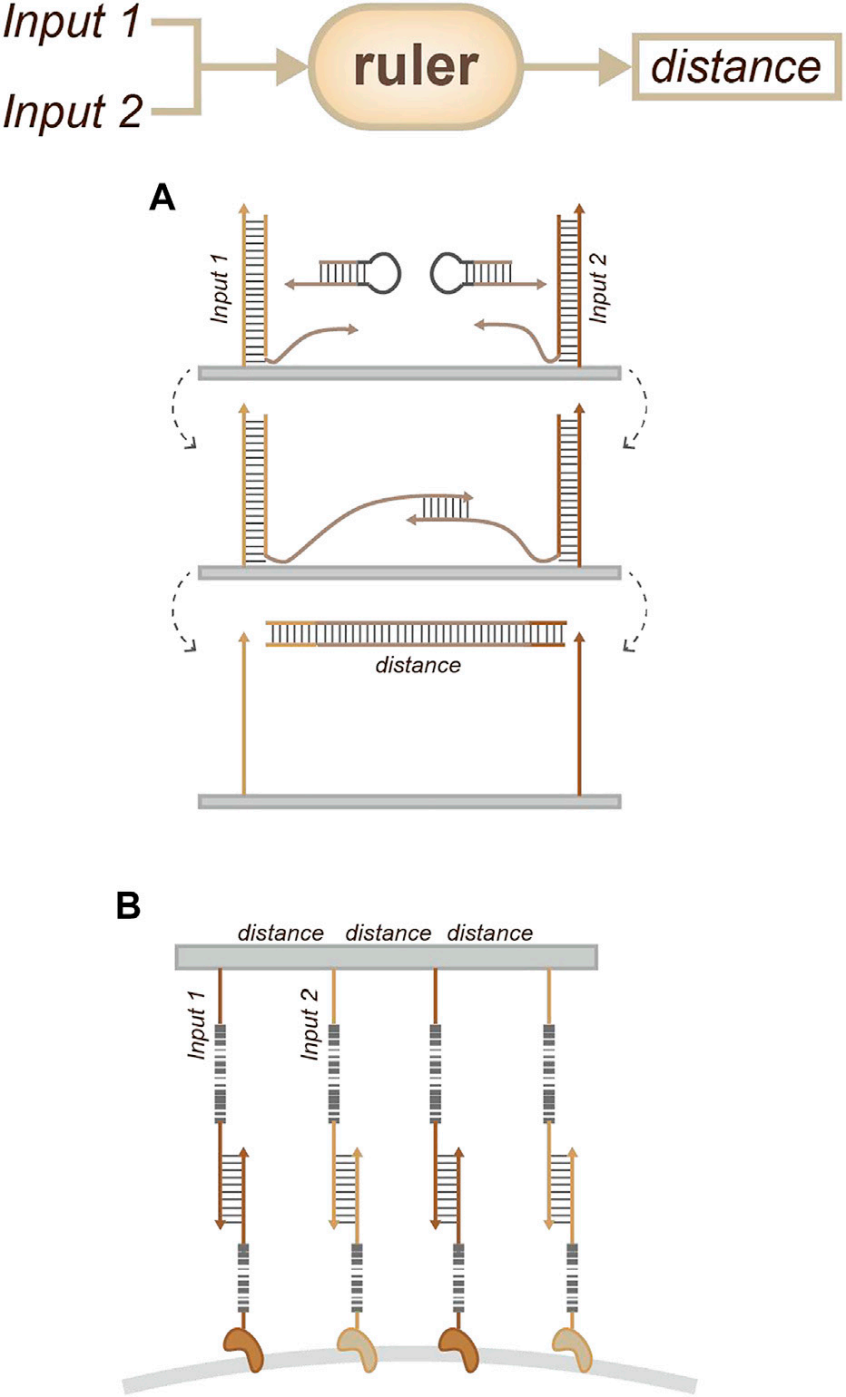
Strategies for location sensing. **(A)** Molecular ruler (Gopalkrishnan et al., 2020), which can measure pairwise distances between labeled sites by measuring the DNA sequence. **(B)** NanoDeep (Ambrosetti et al., 2021), which can transform the organization information of membrane proteins into a DNA sequencing readout.

A similar strategy involves the measurement of pairwise distances between labeled sites. This strategy involves the aid of a corresponding extension hairpin (Figure 10A). Specifically, the target is labeled with DNA barcodes, and users can measure pairwise distances between labeled sites by measuring the DNA sequence that is written between the barcodes (Gopalkrishnan et al., 2020).

Another non-microscopy-based method is called NanoDeep (Figure 10B). This was developed for ensemble analysis of membrane protein nanodomains. This method can transform the organization information of membrane proteins into a DNA sequencing readout using DNA nanoassemblies. It can be used to analyze the inventory of proteins by modular design. Such strategies provide new insights into the composition and spatial organization of molecule nanoenvironments and the role of these in the regulation of molecular function (Ambrosetti et al., 2021).

#### 2.3.2 Potential interactions

Signal transduction in a cell depends on molecular interactions. For example, the interaction between proteins might lead to phosphorylation, ubiquitination, and activation or inhibition of signal pathways. Moreover, the interaction between proteins and nucleic acids might result in transcription or translation. However, these interactions usually happen within a very small space, within a distance of around 10 nm (Bajar et al., 2016). Hence, obtaining information on adjacent targets is important and informative for understanding biological processes.

**FIGURE 11:**
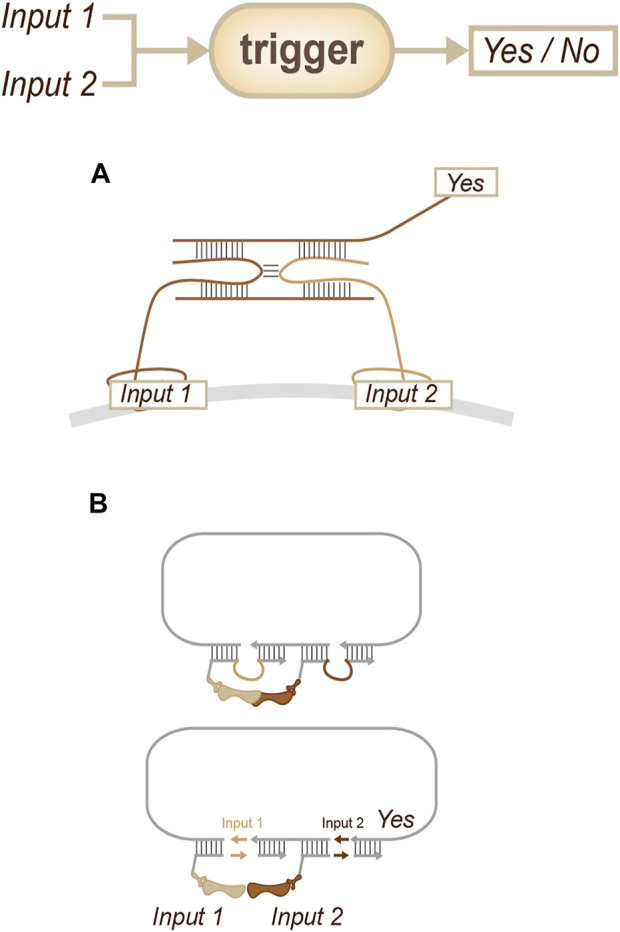
Strategies for potential interaction sensing. **(A)** Information will be output if there is co-recognition. (Chang et al., 2019; Gao et al., 2020). **(B)** Concomitant information will be inserted if there is a potential interaction; with the extension of the sequence, the interaction will be reported. (Raykova et al., 2022).

The general strategy for obtaining this information involves collecting information on a recognition target at a small distance. Researchers have used antibodies or aptamers to sense target membrane proteins and then integrate information on their co-existence (Figure 11A) which hybridization chain reaction (HCR) and rolling circle amplification (RCA) have been chosen to achieved signal amplification (Chang et al., 2019; Gao et al., 2020). In 2022, Söderberg’s group reported a fantastic strategy called MolBoolean (Figure 11B): this is a method that can detect interactions between endogenous proteins in various subcellular compartments. This approach utilizes antibody-DNA conjugates for identification and signal amplification and can provide an indication of the relative abundances of two different proteins or identify that these proteins are close enough to be considered a complex (Raykova et al., 2022). Due to its applicability both in fixed cells and tissue sections, the specific and quantifiable data that this method generates might provide opportunities for both diagnosis and medical research.

## 3 Conclusion and outlook

In summary, this paper has reviewed the recent advances in the use of DNA functional modules in biological systems. With the use of sensors and switches, target identity information can be transformed into DNA oligos: various target-response-based oligos can be exposed or released and target-response-based oligos can be produced. For concentration sensing, thresholds and selectors are common methods for distinguishing concentrations in complex DNA network applications. Strategies for temporal information sensing include order and duration. Sequencers and selectors can be used for order sensing while approaches based on accumulation and consumption can be used for signal delay. Finally, common spatial information sensing strategies include the detection of location information based on organization and the relative position of interactions.

Although there has been great progress in DNA-based functional module design, there are still many challenges. For example, a combined system might contain different functional modules with distinct design patterns, and coupling several functional modules can lead to difficulties such as crosstalk, which can cause errors. Therefore, a standardized, modular, characterized, and orthogonal engineering combined system could make designing easier, faster, and more predictable. Furthermore, given the wide practical applications of functional modules, it is expected that system modeling will be developed to consider the collaboration of multiple modules. Ideally, one could dependably generate an entirely new system with novel functions and predictable behavior from a standardized parts list. Future studies should also focus on developing new sensing strategies and exploring new theories.
